# Long-Term Survival in Nonsurgical Esophageal Cancer Patients Who Received Consolidation Chemotherapy Compared With Patients Who Received Concurrent Chemoradiotherapy Alone: A Systematic Review and Meta-Analysis

**DOI:** 10.3389/fonc.2020.604657

**Published:** 2021-01-07

**Authors:** Xiaojie Xia, Zeyuan Liu, Qin Qin, Xiaoke Di, Zhaoyue Zhang, Xinchen Sun, Xiaolin Ge

**Affiliations:** ^1^ Department of Radiation Oncology, Jiangsu Province Hospital and Nanjing Medical University First Affiliated Hospital, Nanjing, China; ^2^ Department of Radiation Oncology, School of Nanjing Medical University, Jiangsu Province Hospital and Nanjing Medical University First Affiliated Hospital, Nanjing, China

**Keywords:** esophageal cancer, consolidation chemotherapy, chemoradiotherapy, meta-analysis, toxicity

## Abstract

**Background:**

Concurrent chemoradiotherapy (CCRT) is the standard treatment for nonsurgical esophageal cancer (EC). However, esophageal cancer patients receiving CCRT alone are still unsatisfactory in terms of local control and overall survival (OS) benefit. Clinicians generally add consolidation chemotherapy (CCT) after CCRT. It remains controversial whether CCT following CCRT is beneficial for esophageal cancer. We, therefore, undertook a meta-analysis to assess the need for CCT in inoperable esophageal cancer.

**Materials and Methods:**

We combed PubMed, Embase, Cochrane Library, Web of Science, and CNKI for relevant published articles up to July 2020 that compared CCRT plus CCT to CCRT alone for patients with nonsurgical EC. Our primary endpoint was OS and progression-free survival (PFS), and the secondary endpoint was treatment toxicity. We analyzed the hazard ratio (HR) to estimate the time-to-event data and the odds ratio (OR) to compare the treatment-related effect. To assess heterogeneity, we performed the I^2^ test and examined publication bias using funnel plots analysis.

**Results:**

The 11 retrospective studies involved 2008 patients. Of these 2008 patients, 1018 received CCRT plus CCT, and 990 received CCRT. Compared to CCRT alone, CCT after CCRT did not improve disease control rate (DCR) (OR 1.66; 95% CI 0.53–5.15, p=0.384) and objective response rate (ORR) (OR 1.44; 95% CI 0.62–3.35, p=0.393). However, OS (HR 0.72; 95% CI 0.59–0.86, p < 0.001) and PFS (HR 0.61; 95% CI 0.44–0.84, p=0.003) did increase. Our results show that CCT plus CCRT had a clear survival advantage over CCRT alone. The risk of treatment toxicity did not increase for EC patients who received CCT.

**Conclusion:**

CCT after CCRT significantly increases OS and PFS in patients with nonsurgical EC and could provide them remarkable survival benefits. The results provide an evidence-based framework for the use of CCT after CCRT.

## Introduction

Esophageal cancer (EC) is one of the most common malignant tumors of the digestive system. It ranks seventh in terms of tumor incidence and is the sixth leading cause of cancer-related death (1). Esophageal squamous cell carcinoma (ESCC) is the predominant histological type reported in Asian countries although adenocarcinoma is more common in Western countries (2). Most patients with EC are diagnosed in an advanced stage due to a lack of specificity of early symptoms and have lost the opportunity to undergo radical surgery (3). Concurrent chemoradiotherapy (CCRT) is considered as the standard treatment for patients with unresectable EC, especially for elderly patients (4). However, the 5-year survival rate of EC patients receiving CCRT is about 10%–30% due to local tumor recurrence and distant metastasis (5). Therefore, there is need for a more effective method to further improve the survival rate of EC patients who receive CCRT.

As far as we know, there are no large-scale clinical trials to explore the efficacy of consolidation chemotherapy (CCT) after CCRT in EC patients. Studies have confirmed that CCT plays a significant role in the treatment of nasopharyngeal cancer, lung cancer, and other tumors (6, 7). Some studies (8, 9) find that CCT did prolong the survival time of patients with EC although others (10, 11) show that CCT has nothing to do with improving patient prognosis. It is not clear whether CCT can improve the survival rate of EC patients, and there are no relevant and exhaustive studies to determine whether CCT is related to patient prognosis.

CCT aims to inhibit tumor cell proliferation by eliminating subclinical lesions after CCRT. To date, several case-control studies have been published, but no randomized controlled studies have been conducted to explore the effect of CCT on EC after receiving CCRT. The results of each case-control study differ and are not sufficient to detect the role of CCT. In such circumstances, we first performed a meta-analysis to estimate the survival benefit of CCT in EC patients.

## Material and Methods

### Search Strategy

In May 2020 and July 2020, we did two comprehensive searches on the Pubmed, Embase, Cochrane Library, Web of Science, and CNKI databases to make sure we collected all the literature related to CCT of EC. The keywords used for the online search were “esophageal neoplasms,” “concurrent chemoradiotherapy,” and “consolidation chemotherapy.” Apart from searching the databases, we did a manual search for potential studies from the cited documents of the included studies. Two researchers independently carried out the search.

### Study Selection

Studies were eligible if they met the following inclusion criteria: (1) participants diagnosed with pathologically inoperable EC; (2) studies including survival outcomes between the CCRT-alone and CCRT–CCT groups; (3) case reports, reviews, letters, comments, and editorials were excluded; (4) treatment response was evaluated according to Response Evaluation Criteria in Solid Tumors (RECIST), and adverse events were evaluated based on the National Cancer Institute’s Common Terminology Criteria for Adverse Events (CTCAE); (5) hazard ratio (HR) and 95% confidence interval (95% CI) were available directly or indirectly; (6) the language of the included documents was English or Chinese.

### Data Collection and Quality Assessment

Data were extracted from eligible studies based on systemic review, and the meta-analysis was reported according to the Preferred Items for Systematic Reviews and Meta-Analyses (PRISMA) guidelines (12) and the Observational Studies in Epidemiology (MOOSE) guidelines (13). Two researchers independently extracted the following data: author, year of publication, trial region, sample size, number in CCRT-alone group, number in CCRT–CCT group, pathological type, clinical stage, staging standard, follow-up time, univariate or multivariate analysis, survival outcome, treatment regimen, HR and 95% CI, adverse events, and treatment response. If both univariate and multivariate results were available, univariate was preferred for the following reasons. Only 27.3% (univariate=10, multivariate=3, both=2) of all studies report results of multivariate analysis, and none of them describes the multivariate analysis method. The difference in numbers and types of variables entered also increased the bias in multivariate analysis results.

The Newcastle–Ottawa Scale (NOS) (14), which was developed for nonrandomized studies, was applied to assess the studies’ quality based on three categories: selected cases, comparability of groups, and assessment of outcomes. Two researchers obtained independent scores according to the classification prompts for the three categories. Scores ranged from 0 to 9 with higher scores indicating better quality of literature. Studies scoring higher than 6 were considered to be of high quality. Any disagreements regarding study selection, data collection, and quality assessment were resolved through discussion.

### Statistical Analysis

HR and 95% CI were used to assess survival outcomes. The definition of HR was CCRT–CCT group versus CCRT-alone group, and we took the reciprocal of HR and 95% CI in studies whose HR was CCRT-alone group versus CCRT–CCT group. When possible, HR and 95% CI were obtained directly from the studies. HRs were calculated from survival curves in cases in which studies did not report the exact HR values with the methods previously reported by Tierney (15). If 95% CI of HR covered 1, it was considered insignificant. The meaning of HR < 1 was defined as CCT decreasing the risk of death, and HR > 1 indicated CCT increased the risk of death. Response rate and adverse events were assessed by odds ratios (ORs). The definition of OR was CCRT–CCT group versus CCRT-alone group.

I^2^ statistics were used to assess heterogeneity between studies, which estimated the total percentage variation across studies due to heterogeneity rather than chance (16). A fixed effect model was used in the absence of significant heterogeneity (I^2^ < 50%). Otherwise, a random effect model was applied. We also performed a subgroup analysis and a sensitivity analysis to find the source of the heterogeneity. Publication bias was assessed by Begg’s and Egger’s tests (17) and funnel plots. *P* less than 0.05 was considered as existing publication bias. The trim-and-fill method was applied to adjust the HR for publication bias among studies. A two-sided *p* value less than 0.05 was considered statistically significant. All statistical analyses were performed using Stata statistical software 15.0 (Stata Corporation, College Station, TX, USA).

## Results

### Study Selection

**Figure 1:**
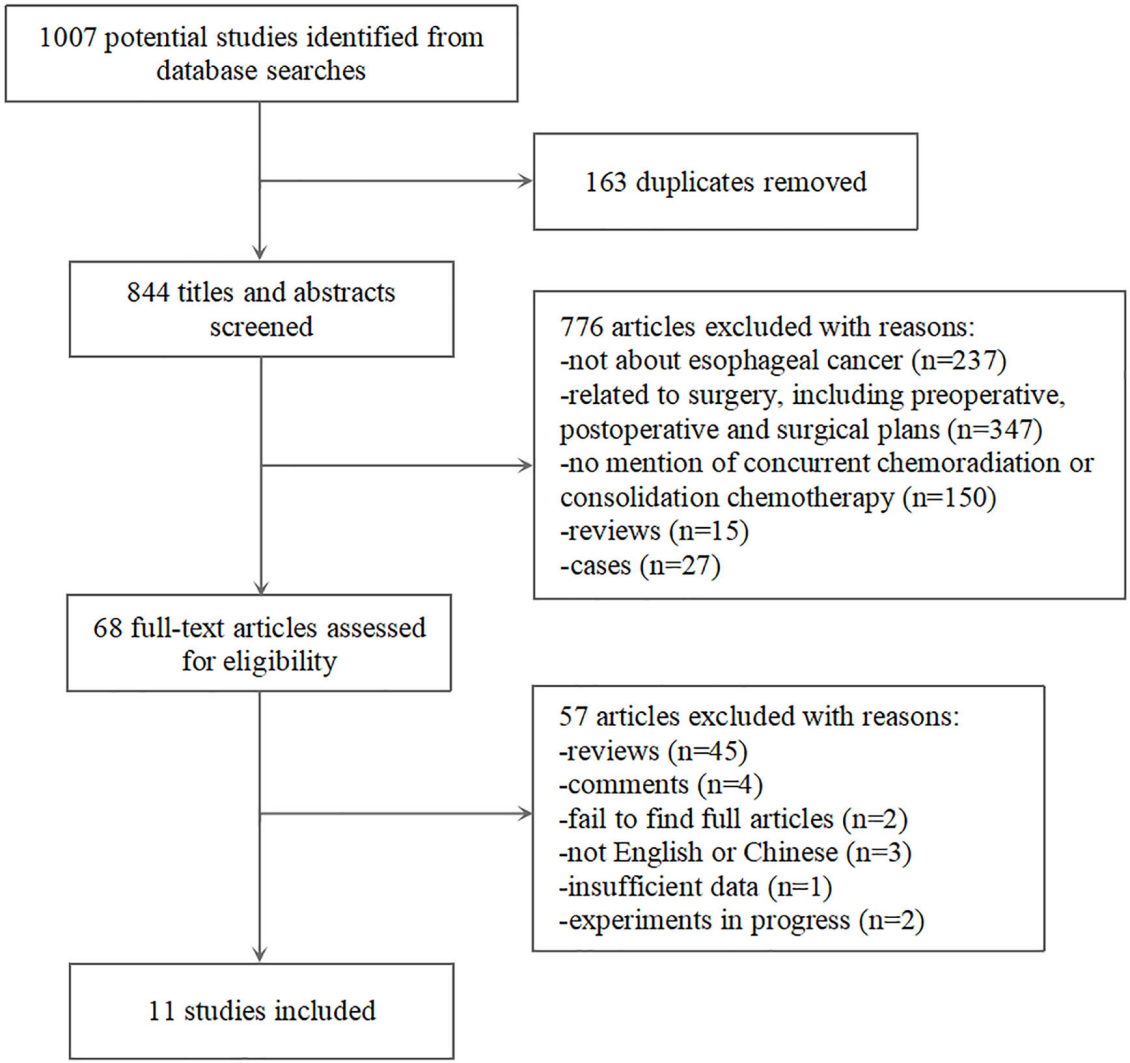
Flow chart of the study selection process.

As summarized in **Figure 1**, 1007 records of relevant studies were obtained from PubMed (n=685) and other databases (n=322). Of these, 68 studies passed the title and abstract screening. After full text screening, 57 studies were excluded for reasons such as lack of relevant data or data duplication. Finally, 11 case-control studies were included in this meta-analysis (8–11, 18–24).

### Characteristics of Included Studies and Quality Assessment

There were 2008 unresectable EC patients in the 11 retrospective trials with 1018 in the intervention groups (CCRT–CCT) and 990 in the control groups (CCRT-alone). The basic characteristics of the included literature and the treatment regimens used are described in **Table 1**. Eligible studies were published in the past 7 years. All 11 trials were retrospective studies from a single center, and participants were from Korea and China. The clinical TNM stage of patients in most studies was diagnosed according to the American Joint Committee on Cancer (AJCC)/Union for International Cancer Control (UICC) TNM staging system. Most of the EC patients participating in the enrolled studies were at an advanced stage except for one study. The stage of patients published by Wu, S. X. et al. were from stage I to III (21). The total radiation dose in the enrolled studies ranged from 40 to 70 Gy in fractionated doses of 1.8 or 2 Gy per day. Synchronized chemotherapy regimens were based on platinum, including paclitaxel combined with platinum or 5-FU combined with platinum. The regimens for CCT were 1–6 cycles of paclitaxel or 5-FU combined with platinum. The estimated NOS scores of all included studies were higher than 5, and the median quality score of included studies was 6.

**Table 1:** Characteristics of included studies.

Author	Year	Region	Sample Size	Number CCRT/CCRT-CCT	Tumour type	Clinical stage	Staging standard	Treatment regimen			Median follow-up period (months)	Survival analysis	Outcome	Quality scores
								concurrent chemotherapy	radiotherapy	consolidation chemotherapy				
Chen, M (11).	2018	China	187	98/89	ESCC	II37/III47/IVA61/IVB42	8th AJCC	PF/TP	40-50.4Gy(1.8-2.2Gy/fractions)	NR 1-4 cycles	20	Univariate analysis	OS/LFFS/DFFS	6
Koh, H. K (18).	2020	Korea	73	17/56	ESCC	NR	NR	PF	50-70Gy(1.8-2Gy/fractions)	PF	13.3	Multivariate analysis	OS/PFS/LFFS	6
Chen, Y (19).	2018	China	524	262/262	ESCC	II218/III306	7th AJCC/UICC	PF: 5-FU (500 mg/m2) d1-d5+ cisplatin(15 mg/m2) d1-d5 q4w	>50.4Gy(1.8-2Gy/fractions)	PF: 5-FU (750 mg/m2) d1-d4+ cisplatin(75 mg/m2) d1 q4w 2cycles	42.5	Univariate analysis	OS/PFS	7
Luo, H (20).	2016	China	79	41/38	Mixed	II28/III51	6th AJCC/UICC	TP: docetaxel (25 mg/m2) d1+cisplatin(25 mg/m2) d1-d3 qw	56-60Gy(1.8-2Gy/fractions)	TP: docetaxel (60 mg/m2) d1,d8+cisplatin(75 mg/m2) d1-d5 q3w 4 cycles	NR	Univariate analysis	OS/PFS	6
Wu, S. X (21).	2017	China	209	142/67	ESCC	I41/II82/III86	NR	PF: 5-FU (7500 mg/m2) d1-d4+ cisplatin(20-25 mg/m2) d1-d3 q3w	>50.4Gy(2Gy/fractions)	(1) PF: 5-FU (7500 mg/m2) d1-d4+ cisplatin(20-25 mg/m2) d1-d3 2cycles; (2) TP:docetaxel (60-70 mg/m2) d1+cisplatin(20-25 mg/m2) d1-d3/nedaplatin (60-70 mg/m2) d1; 2cycles	NR	Multivariate analysis/Univariate analysis	OS/PFS	7
Chen, H (22).	2018	China	124	59/65	ESCC	NR	6th AJCC/UICC	(1)PF: 5-FU (500 mg/m2) d1-d5+ cisplatin(75-80 mg/m2) d1-d3 q3w; (2) TP:paclitaxel (135-175 mg/m2) d1+cisplatin(75-80 mg/m2) d1-d3 q3w	50-74Gy(1.8-2.2Gy/fractions)	based on platinum 2-4 cycles	18.5	Univariate analysis	OS/PFS	6
Zhang, A. D (8).	2020	China	222	109/113	ESCC	NR	7th AJCC/UICC	(1)LPF: 5-FU (450-500 mg/m2) d1-d5+ cisplatin(25 mg/m2) d1-d3 + calcium folinate(200 mg/m2) d1-d5 1-2 cycles; (2)PF: 5-FU (450-500 mg/m2) d1-d5+ cisplatin(25 mg/m2) d1-d3 1-2 cycles; (3) TP:paclitaxel (135-175 mg/m2) d1+cisplatin(25 mg/m2) d1-d3 1-2 cycles	50.4-66Gy(1.8-2Gy/fractions)	(1)LPF: 5-FU (450-500 mg/m2) d1-d5+ cisplatin(25 mg/m2) d1-d3 + calcium folinate(200 mg/m2) d1-d5 1-4 cycles; (2)PF: 5-FU (450-500 mg/m2) d1-d5+ cisplatin(25 mg/m2) d1-d3 1-4 cycles; (3) TP:paclitaxel (135-175 mg/m2) d1+cisplatin(25 mg/m2) d1-d3 1-4 cycles	93	Univariate analysis	OS	7
Kim, D. E (9).	2013	Korea	59	16/43	ESCC	III/IVA	6th AJCC/UICC	(1)PF: 5-FU (1000 mg/m2) d1-d4+ cisplatin(75 mg/m2) d1 2 cycles; (2) TP:docetaxel (20 mg/m2) +cisplatin(25 mg/m2) d1,d15,d18 2 cycles	50.4-64.8Gy(1.8Gy/fractions)	based on platinum 2-6 cycles	18.4	Univariate analysis	OS	6
Li, Y. M (10).	2017	China	102	53/49	ESCC	II41/III61	Analysis on the applicability of the nonsurgical clinical staging for esophageal carcinoma	(1)PF: 5-FU (500 mg/m2) d1-d5+ cisplatin(80 mg/m2) d1-d3 q4w; (2) TP:paclitaxel (135 mg/m2) +cisplatin(75 mg/m2) d1-d3 q3w	50.4-57.6Gy(1.8Gy/fractions)	(1)PF: 5-FU (500 mg/m2) d1-d5+ cisplatin(80 mg/m2) d1-d3 q4w; (2) TP:paclitaxel (175 mg/m2) +cisplatin(75 mg/m2) d1-d3 q3w 1-6 cycles	NR	Univariate analysis	OS/PFS	6
Tian, J (23).	2017	China	68	32/36	ESCC	II46/III19/IVA3	6th AJCC/UICC	(1)S-1: TS-1(50 mg bid) d1-d14 q3w;(2)PF: 5-FU (750 mg/m2) d1-d5+ cisplatin(20 mg/m2) d1-d5 q3w; (3) TP:docetaxel (40 mg/m2)/paclitaxel (90 mg/m2) d1,d8,d15+ cisplatin(40 mg/m2) d1,d8,d15 q4w	60 Gy(2Gy/fractions)	(1)S-1: TS-1(50 mg bid) d1-d14 q3w;(2)PF: 5-FU (750 mg/m2) d1-d5+ cisplatin(20 mg/m2) d1-d5 q3w; (3) TP:docetaxel (40 mg/m2)/paclitaxel (90 mg/m2) d1,d8,d15+ cisplatin(40 mg/m2) d1,d8,d15 q4w 1-4 cycles	20	Multivariate analysis/Univariate analysis	OS/PFS	7
Chen, Y (24).	2016	China	361	161/200	ESCC	II119/III242	7th AJCC/UICC	based on platinum	>50.4Gy(1.8-2Gy/fractions)	based on platinum 2-4 cycles	NR	Univariate analysis	OS	5

CCRT, concurrent chemoradiotherapy; CCRT-CCT, consolidation chemotherapy following concurrent chemoradiotherapy; ESCC, esophageal squamous cell carcinoma; NR, not report. AJCC, American Joint Committee on Cancer; UICC, Union for International Cancer Control; PF, 5-FU + cisplatin; TP, docetaxel + cisplatin. LPF, 5-FU + cisplatin + calcium folinate; OS, overall survival; PFS, progression-free survival, DFFS, distant failure-free survival; LFFS, locoregional failure-free survival.

### Survival Analysis

**Figure 2:**
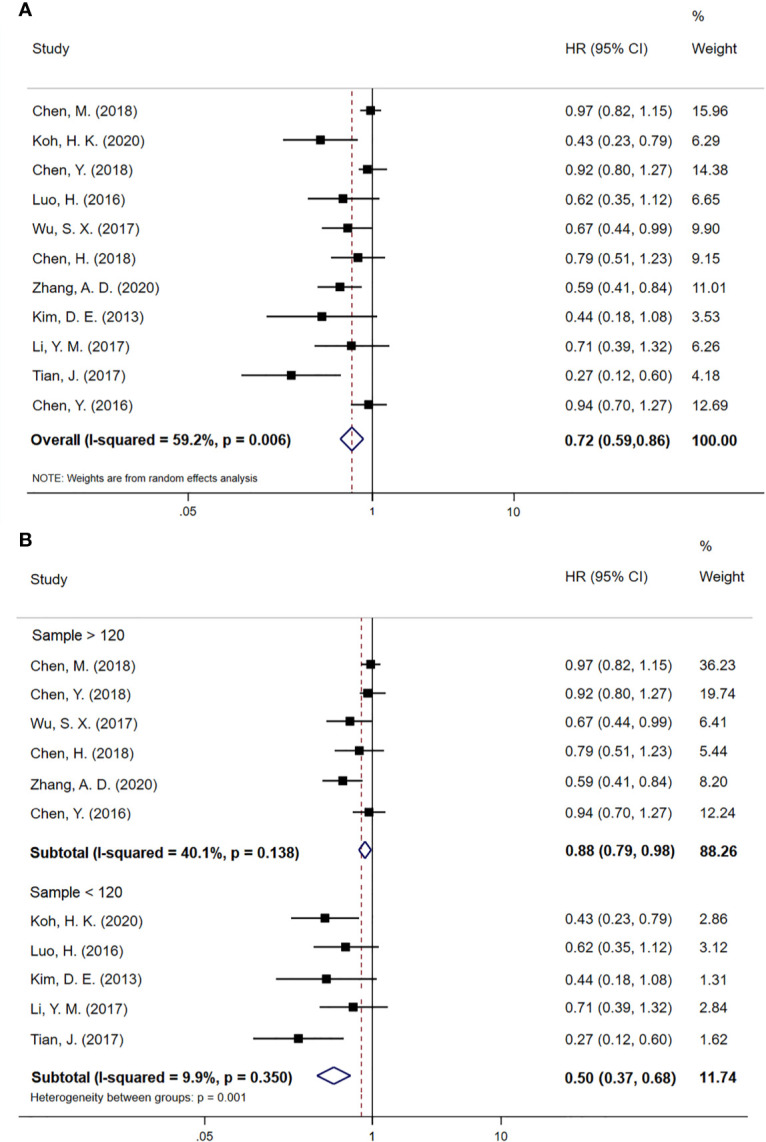
**(A)** Meta-analysis of the associated HRs of OS for CCRT–CCT compared with CCRT alone. **(B)** Subgroup analysis of the associated HRs of OS for CCRT–CCT compared with CCRT alone. HR, hazard ratio; OS, overall survival; 95% CI, 95% confidence interval; CCRT–CCT, consolidation chemotherapy following concurrent chemoradiotherapy; CCRT alone, only concurrent chemoradiotherapy.

We included all 11 case-control studies in the overall survival (OS) analysis, giving 2008 EC patients in total. The forest plot for HR of OS is shown in **Figure 2A**. Patients treated with CCRT followed by CCT had a better survival rate than those treated with CCRT alone (HR 0.72; 95% CI 0.59–0.86, p < 0.001). Statistics suggest that EC patients who have not undergone surgery may benefit from CCT after CCRT. However, obvious heterogeneities were found between studies (P=0.006, I^2^=59.2%). Subsequently, we performed a subgroup analysis based on the sample size of patients with EC. The subgroup analysis results for OS are shown in **Figure 2B**. Nevertheless, six case-control studies with a sample size above 120 (HR 0.88; 95% CI 0.79–0.98, p=0.018) and five case-control studies with a sample size below 120 (HR 0.50; 95% CI 0.37–0.68, p < 0.001) revealed OS was improved with CCT following CCRT compared to CCRT alone. There was no evidence of significant heterogeneity between studies with high sample size (P=0.138, I^2^=40.1%) or with low sample size (P=0.350, I^2^=9.9%).

**Figure 3:**
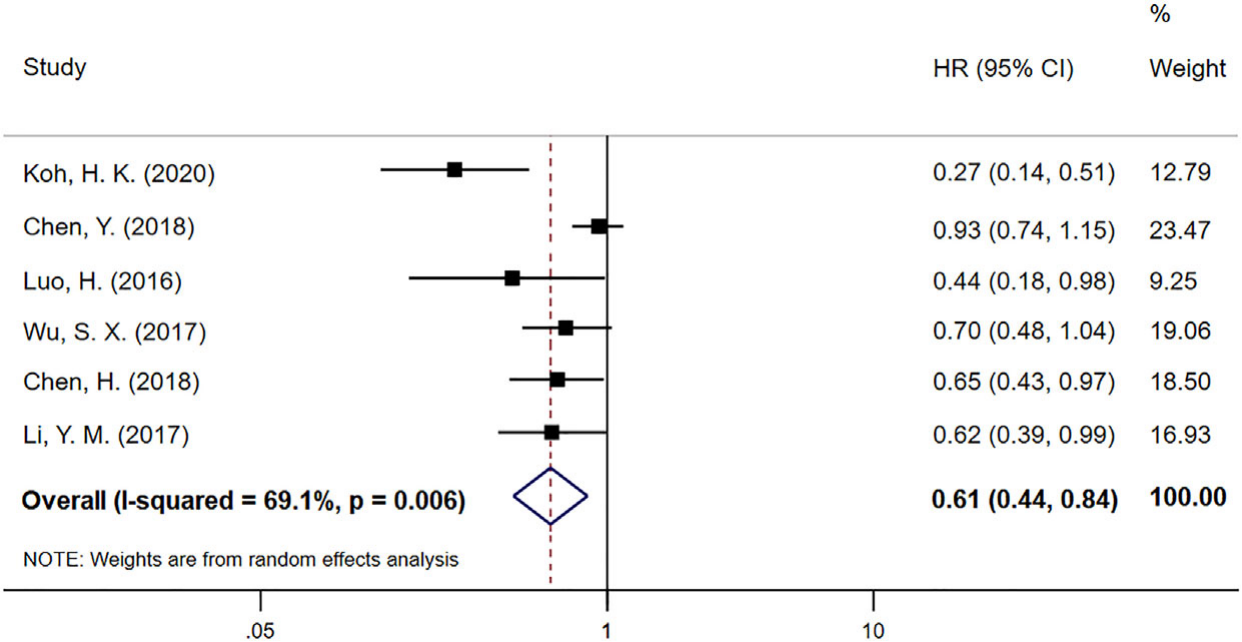
Meta-analysis of the associated HRs of PFS for CCRT–CCT compared with CCRT alone. HR, hazard ratio; PFS, progression-free survival; CCRT–CCT, consolidation chemotherapy following concurrent chemoradiotherapy; CCRT alone only concurrent chemoradiotherapy.

Progression-free survival (PFS) data was extracted from six studies, including 1111 EC patients, in which 537 patients received CCT after CCRT and 574 patients received CCRT alone. The meta-analysis result for PFS is shown in **Figure 3**. PFS in the CCT group was significantly better than that in the CCRT group (HR 0.61; 95% CI 0.44–0.84, p=0.003). There was obvious heterogeneity among these studies (P=0.006, I^2^=69.1%).

In the included studies, only 2 articles reported the survival outcome of locoregional failure-free survival (LFFS). Koh, H. K (18). report that CCT prolonged LFFS, and Chen, M (11). thought there was no difference in LFFS between both groups. Considering the high degree of heterogeneity, no merger was carried out. Chen, M. likewise reports the insignificant result of distant failure-free survival (DFFS).

### Tumor Response

**Figure 4:**
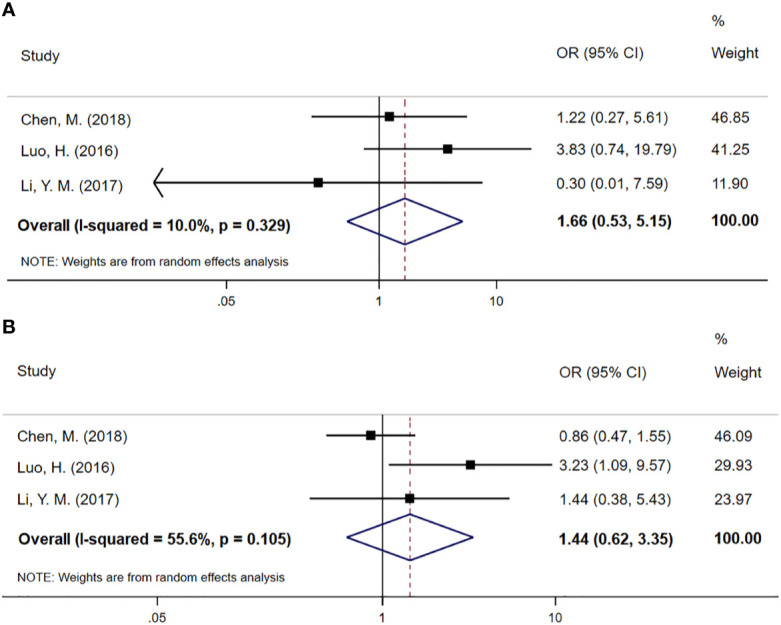
**(A)** Meta-analysis of the associated ORs of DCR for CCRT–CCT compared with CCRT alone. **(B)** Meta-analysis of the associated ORs of ORR for CCRT–CCT compared with CCRT alone. OR, odds ratio; DCR, disease control rate; ORR, objective response rate; 95% CI, 95% confidence interval; CCRT–CCT, consolidation chemotherapy following concurrent chemoradiotherapy; CCRT alone, only concurrent chemoradiotherapy.

Three studies involving 368 cases reported sufficient data on objective response rate (ORR) and disease control rate (DCR). As shown in **Figure 4**, the pooled ORs demonstrate that there was no statistical difference between the CCT followed by CCRT group and the CCRT-alone group (OR 1.66; 95% CI 0.53–3.15, p=0.384 and OR 1.44; 95% CI 0.62–3.35, p=0.393 for DCR and ORR, respectively). No obvious heterogeneity was found in the DCR and ORR analysis (P=0.329, I^2^=10%). Although there were moderate differences in the ORR analysis (I^2^=55.6%), there was no evidence of significant heterogeneity between groups (P=0.105).

### Toxicity

Adverse events occurring during the treatment period were available in only three studies involving 708 patients. Gastrointestinal reactions included nausea, emesis, and anorexia. There were no significant differences between the CCRT–CCT group and the CCRT-alone group regarding hematological or nonhematological adverse events. The risk of adverse event grades of 1–2 and 3–4 were similar. There was no evidence of significant heterogeneity between the trials regarding treatment toxicity. The detailed merger results are shown in **Table 2**.

**Table 2:** Adverse events during the CCRT-CCT or CCRT-alone period.

Adverse events	Grade	No. of studies	No. of patients	Pooled OR and its 95% CI	Meta-regression(*P* value)	Heterogeneity	
						I^2^(%)	*P* value
Leukopenia	0-2	2	178	0.62 (0.26-1.47)	0.28	0	0.80
	3-4	2	178	1.62 (0.68-3.89)	0.28	0	0.80
Thrombocytopenia	0-2	2	178	0.93 (0.18-4.76)	0.93	0	0.42
	3-4	2	178	1.07 (0.21-5.45)	0.93	0	0.42
Neutropenia	0-2	3	702	0.86 (0.59-1.25)	0.42	0	0.89
	3-4	3	702	1.16 (0.80-1.68)	0.42	0	0.89
Anemia	0-2	2	178	0.93 (0.26-3.33)	0.91	0	0.50
	3-4	2	178	1.08 (0.30-3.87)	0.91	0	0.50
Gastrointestinal tract	0-2	3	702	1.35 (0.61-2.98)	0.46	0	0.95
	3-4	3	702	0.74 (0.34-1.64)	0.46	0	0.95
Radiation esophagitis	0-2	3	702	0.94 (0.67-1.31)	0.72	0	0.70
	3-4	2	178	1.84 (0.42-8.01)	0.42	0	0.67
Radiation pneumonia	0-2	3	702	1.05 (0.73-1.50)	0.81	17	0.30
	3-4	3	178	0.71 (0.12-4.31)	0.71	32	0.23

CCRT-CCT, consolidation chemotherapy following concurrent chemoradiotherapy; CCRT-alone, only concurrent chemoradiotherapy. OR, odds ratio; 95% CI, 95% confidence interval.

### Sensitivity Analysis and Publication Bias

**Figure 5:**
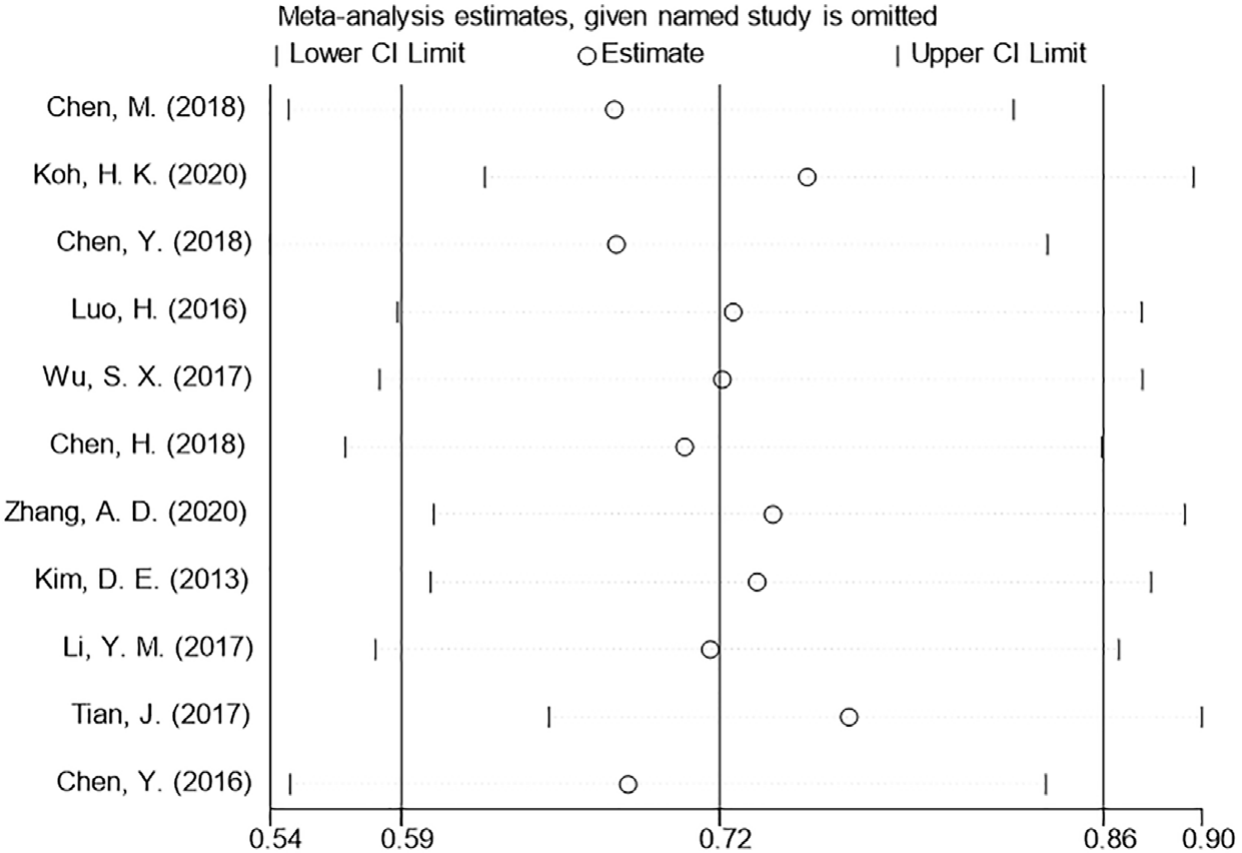
Sensitivity analysis of HRs of OS. HR, hazard ratio.

**Figure 6:**
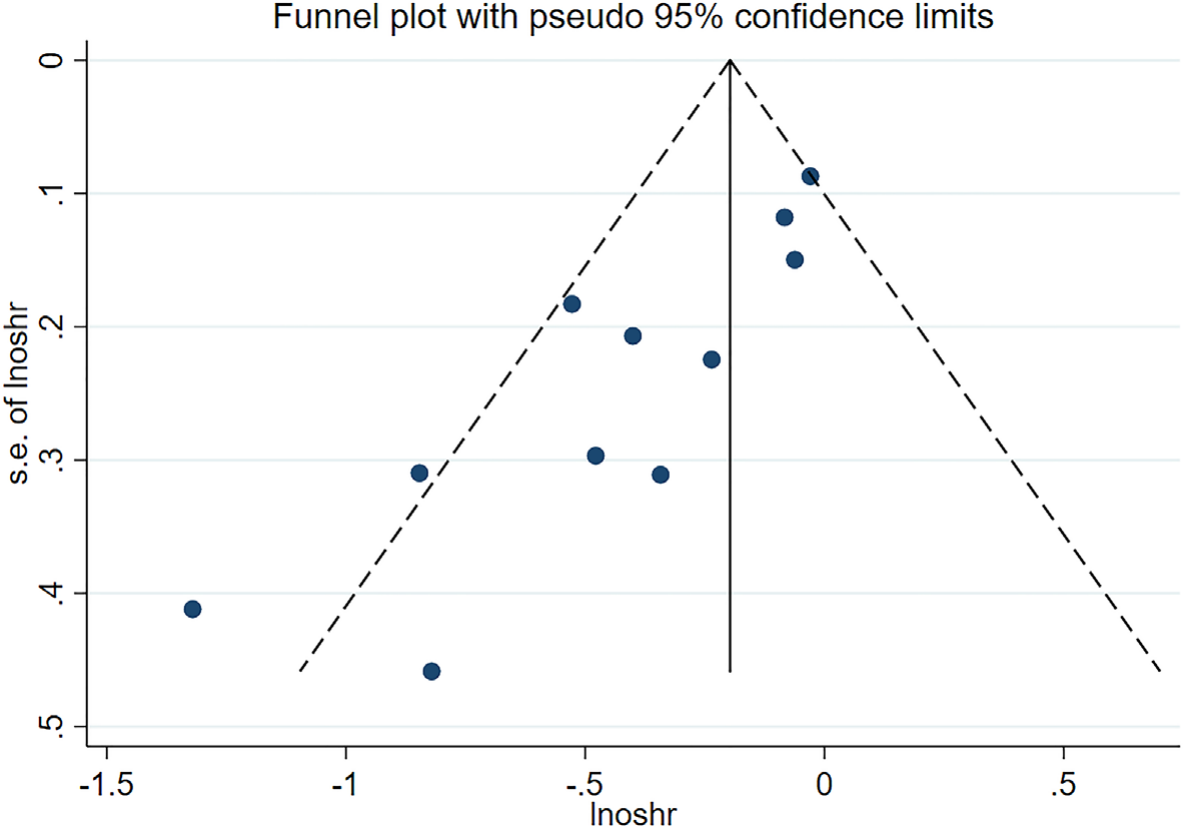
Funnel plot of publication bias for OS.

We used a sensitivity analysis to assess the stability of our overall results. The outcomes of the primary overall analysis were not converted although we removed each study in turn (**Figure 5**). In a pooled analysis of all 11 trials, the funnel plot for OS indicates the existence of publication bias. Two trials were outside the precision line, and one trial was on the line as shown in **Figure 6**. The p values of Begg’s and Egger’s tests (both Ps < 0.05) also indicate the evidence of publication bias. However, further analysis through the trim-and-fill test shows that publication bias did not significantly affect the estimated results (HR 0.72; 95% CI 0.59–0.86, p < 0.001).

## Discussion

Due to the lack of specificity of early symptoms, EC patients are frequently diagnosed at an advanced stage and are mainly elderly patients (25). CRT followed by surgery is considered the optional treatment for resectable EC (26). Patients with late stage or weak constitution generally lose the opportunity to undergo radical surgery. CCRT is the standard therapy for unresectable EC and RTOG 85-01 determines the position of CCRT (27). The 5-year survival rate of EC patients receiving CCRT is still below 30% at present. Clinicians are keen to find optional methods in combination with CCRT to improve survival of EC patients. Because induction chemotherapy before CCRT has been shown to increase the risk of radiation-induced lung tissue damage in EC patients (28), CCT after CCRT has been assumed to improve the therapeutic effect. However, there is still no unanimous conclusion on whether CCT increases the efficacy of nonsurgical EC. In this context, we were the first to conduct this research to estimate the effect of CCT followed by CCRT.

The results of our meta-analysis show that the addition of CCT following CCRT increased OS in patients with nonsurgical EC (HR 0.72; 95% CI 0.59–0.86; p < 0.001). However, the overall result for OS indicates evident heterogeneity (P=0.006, I^2^ = 59.2%). Subgroup analysis based on sample size eliminated significant heterogeneity, and the results of subgroup analysis further confirm this finding. Our sample size is quite large with 2008 patients, and the median NOS score of the 11 case-control studies included is 6, indicating the reliability of our OS results. We further analyzed the data eligible in our articles and found that the clinical features of 7 of those articles are similar in the CCT and the CCRT-alone groups. The clinical features in 4 articles were not detailed (9, 18, 23, 24). The numbers of patients who did not accept CCT after CCRT reported by Koh, H. K. et al (18). and Dae-Eun Kim, et al. (9) are 17 and 16, respectively. Those two articles contained 136 people in total, 103 of whom received CCT. Given that the patients in both articles are late stage and mostly have lymph node metastasis, we found that the number of EC patients with positive lymph nodes receiving CCT is much larger, and this may be an important external factor affecting the results of our meta-analysis. Research has found that EC patients with a poor clinical response to CCRT could benefit from CCT with improved 3-year OS rates in the consolidation group (29). It is known that the clinical response of tumor patients depends largely on the initial stage of cancer. Patients with higher clinical T and N stages generally have a poor response. Those with higher clinical T and N stages have consistently lower pathological CR and OS rates after neoadjuvant CRT (30, 31). Chen Y et al. reveal that the lower esophageal tumor location may have a worse clinical response to CCRT (32). Therefore, we hypothesize that EC patients with high T stage, N stage, and lower tumor location have a poor response to CCRT and may be prone to benefit from CCT. Consistent with our hypothesis, stage III non-small cell lung cancer patients with a good response to CCRT did not benefit from CCT after CCRT (33).

CCT is complementary to synchronous chemoradiation and has a continuous cytotoxic effect on subclinical lesions that cannot be eliminated by CCRT to inhibit tumor cell proliferation (20). It primarily removes cancer cells remaining in the blood to prevent distant tumor metastasis. We hypothesize that this is an intrinsic factor that enables CCT after CCRT to improve patient survival. Because 10 of the 11 articles were limited to squamous cell carcinoma, we did not perform a subgroup analysis based on pathological types of EC. In our meta-analysis, 1111 patients in 6 included articles demonstrated that CCT followed by CCRT can prolong PFS of EC patients (HR 0.61; 95% CI 0.44–0.84; p=0.003). Except for trials conducted by Chen, Y. et al (24). and Wu, S. X. et al. (21), the other 4 trials reported positive PFS results. The results reveal that there was no significant difference in DCR (OR 1.66; 95% CI 0.53–5.15) and ORR (OR 1.44; 95% CI 0.62–3.35) between the CCRT–CCT and CRT-alone groups. Because both results only include 3 experimental results, so the sample size is small and has some degree of heterogeneity, we consider the reliability of these results to be low, and additional research should be required for further analysis. Fortunately, a prospective, open-label, multicenter, randomized, and controlled Phase III trial comparing CCRT plus CCT to CCRT alone for locally advanced ESCC is ongoing in China (34).

The main chemotherapy regimens used in the included studies were docetaxel plus cisplatin (TP) and 5-FU plus cisplatin (PF), and there was a trend in favor of cisplatin-based therapy. However, we were unable to reach a consensus to recommend any chemotherapy regimen due to the limited number of articles exploring a specific chemotherapy regimen, and the patients involved in these studies showed considerable heterogeneity. The chemotherapy regimen in CCT is generally consistent with CCRT in our included research. A published phase III clinical trial shows the 3-year OS of the cisplatin plus fluorouracil regimen was essentially higher than that in the RTOG 8501 trial (51% vs. 30%), and the paclitaxel plus fluorouracil regimen was not superior in terms of OS compared to the standard cisplatin plus fluorouracil regimen in CCRT for patients with locally advanced EC (35). The prevalence of the use of paclitaxel-based regimens for CCRT in EC patients was due to the higher rates of pathologic CR compared to the use of the cisplatin plus fluorouracil regimen (35–37). However, paclitaxel-based regimens in retrospective studies showed an increased risk of radiation pneumonitis in CCRT (38, 39). To date, the cisplatin plus fluorouracil regimen has remained the standard regimen in EC patients, and future clinical trials should focus on finding the optimal chemotherapy regimen.

The pooled ORs of adverse events involving 708 patients in three trials reveal that CCT did not increase treatment toxicity. The main chemotherapy regimen used in the research was paclitaxel combined with platinum or 5-FU combined with platinum. Fluoropyrimidine plus platinum is the standard chemotherapy regimen in East Asia, and 5-fluorouracil, cisplatin, S-1, and docetaxel are chemotherapy drugs commonly used to treat esophagogastric cancer (40). The study of Zhu, Y. et al (41). shows that CCRT with docetaxel plus cisplatin had comparable OS and PFS to CCRT with the 5-Fluorouracil plus cisplatin regimen. Each of these 3 studies (10, 18, 20) shows that CCT can prolong patient survival time without increasing treatment-related toxicity, and the results of the data aggregation in our meta-analysis are consistent with their results.

Our meta-analysis provides favorable evidence on the benefits of CCT followed by CCRT, but our study has several limitations. First, because the articles included are retrospective studies, some biases inevitably generate steps in data integration. Second, some literature does not directly provide HR, and we obtained related data using the method suggested by Tierney (15). These values may differ slightly from the actual values. Third, there is obvious heterogeneity among some results, but this cannot be eliminated by certain methods, such as subgroup analysis, etc. Finally, our meta-analysis shows some publication bias because articles with positive results are easily accepted. Fortunately, publication bias was not significantly affected by the trim-and-fill test, and the sensitivity analysis demonstrates the stability of our results.

## Conclusions

In conclusion, the limited published data demonstrate that the addition of CCT could be of significant benefit in terms of survival in nonsurgical EC cases receiving definitive CCRT. At the same time, the toxicities of therapy are similar between the CCRT–CCT and the CCRT-alone groups. More clinical studies, especially large, randomized, controlled trials are warranted to assess its effectiveness and identify patients who could benefit from CCT. We are looking forward to finding more effective methods to prolong the survival rate of nonsurgical EC patients.

## Data Availability Statement

The original contributions presented in the study are included in the article/supplementary materials. Further inquiries can be directed to the corresponding authors.

## Author Contributions

XX: Roles/writing—original draft, data curation, investigation, methodology. ZL: Roles/writing—original draft, resources, formal analysis, methodology. QQ: Roles/writing—original draft, software, investigation, methodology. XD: Roles/writing—original draft, validation. ZZ: Roles/writing—original draft, visualization. XS: Writing—review and editing, conceptualization, project administration, funding acquisition. XG: Writing—review and editing, conceptualization, supervision, funding acquisition. All authors contributed to the article and approved the submitted version.

## Funding

This work was supported by the National Natural Science Foundation of China [grant numbers 81874217, 81703027, 81703028, 81672983], Young Medical Key Talents of Jiangsu Province [grant number QNRC2016572], Joint Funds for the innovation of science and Technology, and the Priority Academic Program Development of Jiangsu Higher Education Institutions (PAPD) [grant number JX10231801].

## Conflict of Interest

The authors declare that the research was conducted in the absence of any commercial or financial relationships that could be construed as a potential conflict of interest.
